# Reminiscences of an Army Surgeon

**Published:** 1889-09

**Authors:** William Johnstone Fyffe

**Affiliations:** Deputy Surgeon-General, President of the Branch


THE BRISTOL

Medico-Chirurgical Journal.

SEPTEMBER, 1889.

### REMINISCENCES OF AN ARMY-SURGEON.

An Address delivered at the

Annual Meeting of the Bath and Bristol Branch of the

British Medical Association, June 26th, 1889,

BY

WILLIAM JOHNSTONE FYFFE, M.D.,

*Deputy Surgeon-General, President of the Branch.*

DEEPLY as anyone placed in my position by the kindness of his professional brethren may feel the honour and distinction conferred upon him, he is in some respects an object of sympathy, if not of pity.

The search for a subject on which to base an address; the certainty that there lies at his disposal not an inch of untrodden ground; the hopeless conviction that there is nothing new under the sun; the dread of exhausting the patience of his audience, or sending them into a merciful condition of peaceful unconsciousness—all crowd into the mind of him who sits down to compose a presidential address.

But, as Sydney Smith observes, "To do anything in this world worth doing, we must not stand back shivering and thinking of the cold and danger, but jump in and scramble through as well as we can." Despairing of being able to find a subject of medical science on which I might venture with any degree of confidence to address you, and having an antipathy to matters of medical ethics or polemics, I shall not attempt to speak on any of these questions.

But reflecting on the fact that my professional life has lasted for over forty years—the greater part of it having been passed as an army medical officer—I thought I might possibly interest you a little by reviewing some reminiscences of bygone days, even if I should lay myself open to the charge of fighting old battles over again.

Forty-four years ago England had been thirty years at peace. The Battle of Waterloo closed the great campaign of Wellington in 1815. Some smaller wars subsequently occurred in the defence of our Colonial Empire, and on the north-west frontier of India; but no great war called out the latent energies of Britain until the Crimean War of 1854. In 1842, it is true, the massacre of the whole of General Elphinstone's army in the Khyber Pass shocked England. Only one man escaped—Dr. Brydon, the surgeon of the 44th Regiment, who, after great suffering, reached the fortress of Jellalabad, the only survivor of a force of 12,000 persons, including camp-followers, who had marched from Cabul. This episode in the life of an army-surgeon has been made the subject of Miss Thompson's celebrated picture, "The Remnant of an Army."

At the time I entered Her Majesty's service, in 1848, our profession was over-stocked. For men who had no private means, it was difficult to find suitable employment either civil or military. Professional status was at rather a low ebb. The Director-General of the Army Medical Department at this time, an officer who had done good service, but who had held the appointment for thirty years, used to declare that he could if he chose obtain medical officers for half-a-crown a day. Possibly this was an exaggeration; but students were considered fortunate if they could obtain the promise of a commission, a boon then only to be had by nomination and interest.

In my own case, I had to wait for nearly two years before my turn came, so many were the applications to serve Her Majesty for the yearly sum of £126, and free quarters.

There was no Army Medical School then in existence. The only special instruction given, so far as I am aware, was a course of lectures on Military Surgery, given by Sir George Ballingall, in the Edinburgh School of Medicine, and later on a similar course by Mr. Tuffnell of Dublin. There was, however, a fair supply of good literature on the subject, notably the elaborate works of Robert Jackson, probably the greatest thinker for the soldier's good who ever lived, excepting only the late Professor Parkes.

The treatment of disease by the abstraction of blood, either local or general, was then in full force, and every army-surgeon was expected to be an adept with the lancet and the cupping-glass. Special instruction was given to us in the latter operation by a professional cupper in London, named Mapleson, who, I fancy, made a tolerably good income out of the army medical recruit, as we had to pay him a liberal fee. The instruction was peculiar. There was no patient on whom to practice the operation. The subject on which we practised was an old leather-covered sofa, and I well remember the feeling of satisfaction which passed through my youthful bosom, when I was enabled, by an act of prestidigitation more easily imagined than described, to spread over this venerable piece of furniture a prodigious number of cupping-glasses in a remarkably short space of time.

Every medical officer, on entering the service, had to pass a period of probation at the General Hospital, Fort Pitt, Chatham. Here we had to live, at our own cost, at an expensive Mess. But the time was not without its uses. It gave men the opportunity of judging how they would like the army-life, and there were excellent opportunities for professional study and improvement. Besides the practice of the Hospital, there was an admirable library, an excellent pathological museum, and ample opportunities, under a competent instructor, of practising surgical operations on the dead subject.

All those in my year elected to remain in the service, and in a few months were scattered over the world—all with one exception, a brilliant young man who had obtained the highest distinction as a student. We formed a friendly liking together. I urged him strongly not to enter the army, pointing out to him that with his attainments he was sure to do well in private practice. My prophecy was realised. He took my advice and resigned. I have never met him since; but he is now, I believe, one of the most successful and distinguished physicians of the Midland Counties, and a Fellow of the Royal Society.

The medical service was then principally recruited from the Scotch and Irish schools, some of them very rough specimens, who were entirely innocent at first of the ordinary uses of a silver fork or finger-basin at dinner, and whose period of probation at a military Mess-table was not the least useful part of their education; but under this rather uncultivated exterior there were not a few instances of those who, in their subsequent career, gave testimony that they possessed a high sense of duty, and a lofty estimate of the purposes of life.

It was generally understood that Scotchmen obtained the best appointments, especially Aberdeen men, as the Director-General hailed from the granite city. I had a Scotch name and parentage, but did not hold a Scotch degree, and in consequence found myself gazetted to the worst appointment in the service, the Assistant-Surgeoncy of the 3rd West India Regiment. We received the first intimation of the corps to which we were destined, not from the War Office officials, but from persons much more important in our eyes; namely, our respective tailors and outfitters. These gentlemen had the War Office messengers in their pay, and they obtained in this way the first look at the *Gazette* on its way to the printing office. Armed with this information, the tailors came to Fort Pitt, and as their reward for imparting to the anxious candidate the news of his destination, claimed the privilege of supplying him with his uniform and outfit.

My appointment to a black regiment was a heavy blow to me; but as I had determined to take the rough with the smooth in life—and, indeed, having no other alternative—I accepted it, and, like many other events in life's journey which look dark at first, it had its bright side.

My period of service in the West India Islands was profitable in one sense, and instructive in another. I obtained a good private practice, and I became familiar with the clinical features of those formidable fevers, only to be seen in the tropics. It also brought me into contact with an epidemic of Asiatic cholera, perhaps one of the most destructive that ever visited any part of the world. A West Indian town without drainage of any sort, supplied with contaminated well-water, is just the field where cholera would rage unchecked. The mortality was appalling. Nearly 90 per cent. of those attacked died. The cases occurred principally among the native population, who were stricken and demoralised by panic.

I learned one or two things in this epidemic. First, that cholera in its epidemic movement, follows the great lines of traffic and human intercourse; that it *does* seek out man wherever he congregates; and that he carries it with him from congregation to congregation.* I have met with it in various parts of the world since, and on every occasion that law of its progress has been indelibly impressed on my mind. I learned also never to despair of the worst case, and to treat actively the preliminary diarrhœa where it exists; but I know of no specific remedy for this disease, and I doubt if our treatment of it has advanced much in the last fifty years.

But it is a different thing when we look at the prevention of cholera by the vast sanitary powers we posssess in this country now. We know how our late distinguished health-officer stamped out the disease from Bristol in 1866, and delivered the city from impending danger more than once; and I do not believe cholera will ever make head in this country again, while our health-officers are on the alert, and attack it without hesitation on its first appearance.

At the beginning of my life in the army, there were

* Maclean *on Tropical Diseases.*

two rather peculiar characters, who were members of the Medical Department. One resided at Chatham—a short stout man, with a sharp peremptory manner, and very fierce look. This was by general report believed to be the original from whom Dickens drew his well-known character of Dr. Slammer, who, as the readers of *Pickwick* will remember, challenged the unfortunate Mr. Winkle in mistake for Jingle, after the Rochester ball. I knew this gentleman well: he was a kind, hospitable, warm-hearted man; but he was very tetchy on the subject of his having heen immortalised by the great novelist. Every one knew of it; but no one dared to call him Slammer, as the penalty might have been serious.

The other had a very remarkable and romantic history. In appearance he was a pale little man, with a very shrill voice. He was a perfect fire-eater. He had fought several duels. He was a person of considerable ability. He passed through a long service of over thirty years, reached the highest rank, and served with honour and distinction in various stations abroad, and he was regarded with great respect by those who were brought into official or professional relations with him. There were queer suspicions and whispered stories about him. His contemporaries did not know quite what to believe. He reached a good old age, unmarried; died; and left particular directions that his body was not to be examined. But, somehow, the murder came out. His body *was* examined, and this distinguished medical officer was discovered to be—a woman!

In 1854 the great Crimean War burst upon us. It found us living in a fools' Paradise. Everything looked perfect on paper, and on parade. The discipline and *physique*, all that dazzles the eye was faultless as regards our troops. The army of Lord Raglan was reviewed by the Sultan on the plains of Scutari, in May of that year. A more splendid host for its size was never seen. But in seven months later, owing to the utter inefficiency and breakdown of the departments of transport and supply, all that was left of that army consisted of a few ragged, miserable, but still heroic soldiers living in wretched tents or in holes in the ground on the bleak hillside before Sebastopol, and a vast majority of emaciated, sickly, diseased men, crowding the wards of Scutari Hospital, with all its filthy horrors so often alluded to in the history of that time.

You will remember that previous to the invasion of the Crimea, the British and French armies occupied Bulgaria—to us an unknown country, unknown, I mean, in reference to its salubrity: we knew nothing of its endemic diseases, nor did our authorities know the most healthy or unhealthy localities. We were not long there before we discovered these things for ourselves. Intermittent and remittent fevers, and dysentery, soon disclosed to us that we were living in the very atmosphere of malaria. Many of our men and officers succumbed or were invalided home. To crown all, cholera came to us from the West, and began slowly, but surely, to spread through our ranks. The onset of these diseases, coupled with that period of inaction and suspense so trying to soldiers in the field, combined to affect seriously the health and *morale* of the army, so that it was with a feeling of relief amounting to enthusiasm that the order to invade the Crimea was received; but there is no doubt, as Sir William Aitken has so clearly pointed out, that this period of four months' encampment in the malarious regions of Bulgaria laid the foundation for the disasters from sickness and death which nearly destroyed the British army in the first winter before Sebastopol.

The victory of the Alma, which caused such enthusiasm in England, and such dismay to Czar Nicholas, taught us our first bitter medical lesson. There was no want of medical officers; but surgical appliances, means of transport, everything needed for the treatment of sick and wounded men, were miserably, hopelessly deficient. I had in my regiment, on the morning after the action, some 70 wounded men and officers, besides several cases of cholera. The ships for transporting the sick and wounded were five miles away by land-journey, lying off the mouth of the Alma river, and for the conveyance of these helpless men I had two hand-stretchers. It is a matter of history that we were indebted to the French army for the conveyance of our wounded to the sea-beach. They had ambulance-carriages and mule-litters, and they helped us nobly; but it took two days to collect our wounded and transport them, a duty which ought to have occupied only a few hours. At last the dismal duty was accomplished, and 2500 sick and wounded men were embarked and sent off to Scutari.

All the wounds in the battle were gun-shot wounds; there were no bayonet or sword wounds, as the opposing forces never came into close contact. All operations were performed with the patients lying on the ground, and mostly without chloroform. A most unhappily worded order from the chief medical officer prevented many from using it. Indeed, I fear the supply was very limited.

There were not wanting instances at this time of real heroism among the members of our profession. It was here, after the retreat of the Russian army, leaving the greater part of its wounded upon the field, that Assistant-

Surgeon Dr. Thompson volunteered to take charge of the whole of the Russian wounded prisoners. Attended only by his soldier-servant, surrounded by scores of enemies, in a hostile country, these two brave men remained at their post, tending with assiduous care their suffering foes, remembering only the great duties of our common humanity. Dr. Thompson only survived a few weeks longer; he fell a victim to cholera, which still steadily but surely dogged our march to Sebastopol. But his act of devotion is remembered to this day in his northern home, where a monument to his memory stands in his native town.

It was here also that Mackenzie, a brilliant young Scotch surgeon, who had joined the medical service as a volunteer, worked unceasingly night and day to help the wounded. He, too, died of cholera a few days later. He had won the affection and respect of all officers and men who had come under his skilful hands, and he died, lamented by the army, a martyr to duty.

The miseries and discomforts of the first winter before Sebastopol are familiar to all who have read Kinglake's unrivalled description, or Russell's masterly correspondence. A series of disasters fell upon the army in rapid succession. The battle of Inkermann, brilliant victory as it was, terribly disabled it. Even at this early period, disease was making daily ravages. Some regiments were reduced to a handful of men; brigades and divisions were the mere shadows of what they had been a few months before. The prospects were gloomy in the extreme. The Light Cavalry division was nearly annihilated by that brilliant charge which poets have lauded, and where every man was a hero, but which in its results was a miserable fiasco, a mere holocaust, brought about no one knew how, by the folly and pride of men who appear to have lost their heads and their tempers at the same time.

The hurricane of November the 14th coming very soon after, left the camp a scene of desolation and misery indescribable; it destroyed several of our ships, notably the *Prince*, containing a great part of the winter-clothing for the army. Confusion reigned supreme; order seemed to be nowhere, except where actual fighting was concerned. For while the departments of supply and transport were hopelessly broken down, still the long line of intrenchments before the besieged city was manned day and night by half-starved and wretchedly-clad men, facing the incessant fire from the Russian batteries, and the icy blasts, still worse to endure, from their terrible climate.

The duties which fall to the lot of army-surgeons after a general action are of a very trying description; while the conquering force moves on, the surgeon is left on the field to exercise his office amidst the most appalling scenes of human suffering. His first feeling is one almost of bewilderment, for he hardly knows with whom to begin, when so many need his help and are crying for it.

The operation of attending to, collecting and removing into a place of safety, a large mass of wounded thus becomes a matter of serious concern.* Much is involved in it: the immediate saving of life endangered by hæmorrhage, the performance of such operations as are absolutely necessary at the moment, and the removal of the wounded in such a manner as may expose them least to those dangers and accidents which may afterwards render life a long misery.

There was no Ambulance Corps in this campaign at

* See *Gunshot Injuries*, by Professor Sir Thomas Longmore, C.B.

first. The duty of carrying the wounded out of action to the rear, where the medical officers were at work, devolved upon the bandsmen of each regiment. Most regimental surgeons have taught these men the use of the tourniquet, and the way of affording rough first aid to the wounded. All these arrangements are now altered, and these duties are done by well-instructed men of the Medical Staff Corps.

I do not intend in this address to speak of gunshot or other injuries received in battle, for I have forgotten much of what I ever knew on the subject; but there are one or two matters of interest, or rather curiosity, to which I may refer.

The term "Windage of Shot" was formerly, even by enlightened observers, applied to those cases in which serious internal mischief has been inflicted without any external marks of violence. They are almost invariably produced by large fragments of shell or round shot.

The injuries* are inflicted instantaneously, and surgeons for many years believed that the rapid flight of the projectile in close proximity to the body caused a sudden impingement of a column of air upon some part of the surface, inflicting rupture and destruction of internal structures without wounding the integument. These views I need not say are entirely exploded, so many instances having occurred of round shot having passed so close to various parts of the body, tearing off the soldier's accoutrements, and portions of his uniform, without inflicting any injury whatever.

The explanation of these cases of so-called windage lies in the direction or obliquity with which the projectile happens to strike against the skin. A round shot in its

* Longmore, *Op. cit.*

flight may, so to speak, roll across the abdomen or the thigh, breaking up and destroying the soft parts or bone, without injuring the integument. Then the elasticity of the integument under certain circumstances is very remarkable. The following case I remember very well to have happened at the Alma. A man of the 42nd Highlanders was struck by a round shot in the abdomen, and instantly killed. After the action was over, a party of soldiers lifted his body for the purpose of burying him, and found that it was unaccountably heavy. The assistant-surgeon of the regiment, Mr. Mackinnon, examined the abdomen: he found a wound, not very large, and on freely opening it up, he discovered a 24-lb. solid shot lying in the cavity among the intestines. The wound in the abdominal wall was much smaller than the diameter of the shot, which is five inches. The spine was ground to pieces, and when the man was lifted, the mass of iron bulged out from his back; but there was no wound there, and the abdominal wall in front prevented the shot from falling out. No doubt it was a spent shot.

The diseases from which the besieging army suffered so terribly were: cholera, dysentery, fever both typhus and typhoid, and different forms of scurvy. These diseases, with the exception of cholera, were the necessary outcome of the conditions under which the army lived. Exposed as they were to intense cold in summer-clothing, fed on salt rations for the most part and biscuit, without fresh vegetables, lime-juice, or any anti-scorbutic, the troops were reduced—until help came—to a condition which forced the *Times*' correspondent to say of them that "the wretched beggar who wanders about the streets of London in the rain led the life of a prince compared with British soldiers who were fighting for their country before Sebastopol. All suffered equally, officers and men. Some of the sons of the best blood in England lived lives in the first winter which the meanest pariah would not have envied ; and yet they never for a moment gave way to despair, or allowed a thought of eventual failure in the great tragedy before them to enter their minds." *

The means of treating the sick at this time were deplorably insufficient. The supply of medicines failed for a time almost entirely, and the only food available was totally unsuited to men suffering from acute disease. All that medical officers could do was to send their patients with as little delay as possible on board the transports for deportation to Scutari. This condition of things lasted for several months, until England was aroused to the condition of her army, and then there came trom the hands of the rich, and the cottages of the humble, such a wealth of generous kindness, that not only all the necessaries of life, but many of its luxuries, were poured out with lavish hand.

But the diseases I have enumerated spared none. Ship after ship carried their sad freight to the Bosphorus and Scutari, and the cemeteries around that lovely Asiatic shore, in sight of the Golden Horn and the mosques and minarets of Constantinople, attest, to the present hour, how British soldiers, from the highest to the lowest rank, laid down their lives for Queen and country.

The extraordinary immunity of the wounded in this war from its two formidable complications—viz., tetanus, and hospital gangrene—has been the subject of frequent remark.† Only three cases of tetanus occurred among the English troops before Sebastopol, and there were very few of gangrene. I had, however, a personal experience

* Russell's Letters. † See Longmore, *Op. cit.*

of hospital-gangrene, a few months later, on the occasion of a voyage from Constantinople to England in medical charge of a sailing transport in which there were 212 wounded men, who had partly, but not wholly, recovered. The voyage occupied 64 days, and we met with most tempestuous weather both in the Mediterranean and Atlantic. The ship was fairly supplied with comforts, but the main deck of a transport in a winter-storm, filled with sick and wounded men, with the hatches battened down, is a fruitful field for the development of germs. We did not know much about germs in those days, and antiseptics were quite unknown. In addition to the wounded, I had cases of cholera and dysentery on board, and before very long hospital-gangrene made its appearance. I do not know any description of this now almost unknown disease which equals that of Hennen.

"A wound," he says, "attacked by hospital gangrene in its most active and concentrated form, presents a horrible aspect within forty-eight hours. The whole surface of the wound has become of a dark-red colour and ragged appearance, with blood partly coagulated and putrid, adhering at every point. The edges are everted, the cuticle separated three-quarters of an inch round, with a concentric circle of inflammation extending beyond it. The limb is swollen for some distance, of a shining, white colour, the whole of it being œdematous and pasty. The pain is burning and unbearable in the part itself, while the extension of the disease in a circular direction may be marked from hour to hour, so that within twenty-four or forty-eight hours the whole of the calf of a leg, or the muscles of the buttock, may disappear, leaving a great hiatus of the most destructive character, exhaling a peculiar stench which can never be mistaken, and spreading with a rapidity quite awful to contemplate. The great nerves and arteries yield last, but they finally give way, closing the scene after repeated hæmorrhages by one which proves the last solace of the unfortunate sufferer."

This description I saw literally fulfilled in the voyage I am speaking of: wounds almost healed, when attacked, presented in a few hours the appearances just described. The whole buttock was destroyed in one case in forty-eight hours, and was only stopped by fuming nitric acid. In another the gangrene attacked a wound near the elbow, rapidly destroying the structures on the inner side of the arm, and opening the brachial artery, which had to be ligatured. Six or seven cases died, but as the weather improved, I was able to get more ventilation into the main deck, and the disease ceased.

During the siege a surgical operation was performed successfully, which I believe was done for the first time by any army-surgeon. This was excision of the head and neck and part of the shaft of the femur for a gunshot fracture. I saw this patient after his return to England. The limb was not a very useful one, but the operation had saved the man's life. By order from the War Office, I took the patient to London to show him to Guthrie, then sinking into the grave, who told me he had advocated the operation many years before. The operation in this case was performed by my friend Dr. O'Leary, who retired from the service, as a Surgeon-General, a few years ago. He was a member of this Branch, and died in Bath in 1886.

There is a remarkable and exceptional pathological fact * which is exhibited more strikingly on the battle-field than anywhere else; and that is, the perpetuation of attitude

* Longmore, *Op. cit.*

and facial expression met with in some soldiers who have been killed by gunshot wounds. Sir Thomas Longmore says: "The attitude of life, the very act which was in progress of performance, the expression of countenance indicating the feelings under which the action was performed, all arrested by the fatal shot, are perpetuated in death. The breathing passionate actors have been instantaneously, as in the fable of Medusa, converted into lifeless fixed statues." I have seen men on the field lying with their rifles in their hands either aiming or preparing to aim at an object, but stone dead. My friend Surgeon-General Mackinnon was one of the first to enter the Redan after it had been evacuated by the Russians, and one of the first things he saw was a Russian officer sitting on a gabion. His arms were folded across his chest, and he appeared to be sleeping; but he was dead, from a rifle-ball through his left breast. There was nothing to show whether he had sat down on the gabion after being shot, or whether he happened to be sitting there when he was killed. Dr. Rossbach of Würzburg has discussed this question in Virchow's *Archives*. He gives a remarkable instance of a soldier who was killed when in the act of raising a cup to his lips, when sitting among his comrades at breakfast. The fragment of shell tore off his skull, leaving nothing but the lower jaw, and he was found twenty-four hours afterwards in a half-sitting position, still holding the cup with his unsupported raised hand up to his headless lower jaw. Rossbach accounts for these phenomena by rapid *rigor mortis*; but this seems hardly tenable: it is more easily accountable on the hypothesis of sudden and permanent muscular spasm and rigidity.*

* See Longmore, *Op. cit.*

Never was there a time when the soldier was made so much of as during this great siege. His sufferings and privations, his dogged courage, and cheerfulness under the most trying circumstances, were a constant theme of praise among his countrymen at home. In the beginning of the year 1855, the condition of the army evoked so much interest that a Royal Commission, with Mr. Sydney Herbert at its head, was formed to investigate everything connected with the soldier's life. When the state of Scutari Hospital became known in England, the sympathy which it evoked took a practical form, and, in the forefront of the efforts which were made, one figure stood prominently forward, whose large-hearted exertions at the time gained her the gratitude of her country; and although the events in which she took a prominent part are among the things of an almost-forgotten past, although she herself is now an old woman, weakened by years and suffering, yet even now the name of Florence Nightingale calls out our admiration and respect, as we remember how she laboured for the soldiers' good in the days gone by.

Scutari Hospital, as I remember it, was an immense barrack. It had never been built for a hospital at all. There was a smaller building which was the hospital proper. But the great Turkish barrack was used for this purpose when the smaller one was found insufficient. Into this, unsuited in every way for such a purpose, poured ship-loads of sick and wounded from the front. Overcrowding was the result, and the resources of the Medical Department, weakened as it was, were unable to meet the strain, and for a time the condition of things was painful in the extreme.

But help was coming from home. Miss Nightingale came armed with great powers. She had the whole country at her back, and cared nothing for responsibility. She disregarded regulations, and broke through the trammels of official restraint in a way no officer, medical or otherwise, would have dared to attempt. There came also from England a large volunteer staff of medical men, among them our friends Dr. Beddoe of Clifton and Dr. Brabazon of Bath, who did good and faithful service in Scutari, at the front, and at the various hospitals established on the Bosphorus and other places.

At last the war was over. Sebastopol was taken after a most heroic resistance. The army came home and received its honours, and, as a first consequence of all that had happened, public men began to enquire into the entire question of sickness and mortality among troops. That enquiry brought to light the fact that in India alone the death rate amounted to 69 per 1,000 per annum. Then followed the great wave of sanitary reform, which spread far and wide. In India the results have been most satisfactory. The death rate in a few years was reduced from 69 to 18 per 1,000 per annum; and at this moment Englishmen are as healthy in India as they are at home.

In 1856 the first stone of the Royal Victoria Hospital, Netley, was laid by H.M. the Queen. It was opened seven years later, in 1863. The Army Medical School, which had been established at Fort Pitt, Chatham, in 1860, was transferred to Netley, and the work of its four eminent Professors—Parkes, Longmore, Maclean, and Aitken—has been carried on there since by them and by their successors to the immense advantage, as I believe, of the British soldier in all parts of the world. Having been on the staff of that school myself, it would not become me to eulogise it; but I may say this, that after reading the attacks which have been recently made upon it in the public press, I can discover no good reason why it should be abolished, nor on what grounds its detractors seek to deprive the young army-surgeon, at his entrance upon a great number of new and unknown duties, of the valuable instruction he receives there. It has often been the butt of erratic and unscrupulous politicians, who, for the sake of courting popularity and winning a few votes by an ostentatious display of false economy, have sought to destroy an institution established by wise and philanthropic men. But I believe it will still live, and continue to instruct the young army-doctor in the practical duties of the life which lies before him, and mainly in those great questions of hygiene and the prevention of disease with which the name of Edmund Parkes will be for ever associated.

Another outcome of the Crimean War was the reorganisation of the Medical Department. In 1858 a Royal Warrant was issued which gave medical officers a position they never had before—increased rank and pay, and the removal of hardships which had long pressed upon them. It has been well called the Magna Charta of the Department.

Thirty years have passed, and I may say scarcely a shred of that covenant remains. It has been so altered, so shorn of its fair proportions, that it has practically ceased to exist. The Army Medical Service has been during all these years the shuttlecock of politicians, economists, and reformers. Its history would form a volume of painful interest. Sometimes it has been reduced so low in prestige that no highly educated student thought of entering its ranks. At other times it has stood so high in reputation that men with the best qualifications have sought employment in it. Only a few years ago, after the conclusion of the Nile campaign under Lord Wolseley, Dr. Richard Quain delivered an address to the probationers who had completed their course of study at Netley. He described the service, as it then existed, as being in a condition almost of perfection, and these sentiments were re-echoed in the public press; and now once more the barometer has fallen, and the ship is sailing through stormy waters, with rocks on all sides. The unfortunate Medical Department is again undergoing the process of official tinkering under the hands of the inevitable Parliamentary Committee. What the result will be no man can tell.* But, judging from my 40 years' experience of the process, I do not expect much; and if the expressed views of some politicians are carried out, ruin, and not reform, will be the result.

Happily, the great Association to which we belong, and of which we are a branch, extends its protecting ægis far and wide, and shields and succours all its members wherever and in whatsoever circumstances they are found. It has not forgotten the public services. Whatever advantages army-surgeons have been able to obtain have been won for them mainly by the equitable representations of their brethren in civil life. By the rules of the military service to which they belong their lips are sealed. They cannot by any united action place their difficulties before the powers that rule them. But the British Medical Association, by its powerful *Journal*, by its Parliamentary Committee, by its representations in season and out of season, has made its voice heard on many occasions, and

* Since the above was written, Lord Camperdown's Committee has issued its Report and Recommendations. The latter are liberal and good; but as there are six dissentient members out of the eight, it is doubtful if they will be carried into effect.

has demonstrated clearly that the fair and just hopes of a body of nearly 1,000 medical men are not to be lightly ignored or contemptuously set aside. I do not doubt it will succeed in the end. But why should all this agitation be needed? Other professions do not appear to have to fight a continual battle and keep up a constant struggle. Why is it so? Is it some part of that shadow which still rests on our whole profession, and which stands between it and the public recognition and appreciation to which its merits entitle it? Possibly it is so.

That shadow can only be dissipated, and will in the end certainly be dispersed, not only by setting before ourselves lofty aims and high aspirations, but by the brighter shining of the light of true scientific progress—by that living movement which makes the goal of yesterday the starting point of to-morrow.

The march of evolution from distant ages of professional obscurity is a slow one. The process by which medicine, in all its branches, has emerged from the darkness and prejudice of centuries is yet incomplete. Its legitimate position is not yet won. Men, it is true, eulogise our unselfish deeds and labours for the general good of mankind, but look coldly on our attempts to mount the social ladder, to win a place and name in the nation's estimate, or to claim a fair share in the distribution of those public honours and rewards which are freely bestowed on other professions and callings in life. Happily, there *is* a popularity which is worth aspiring after, and which is ours alone. It is won in the bosom of families, at the bedside of the sick and suffering—won by the hospital-surgeon and physician from the lips of Christ's poor, and by the hard-worked country practitioner riding through the snow to save the life of some mother in the throes of childbirth.

It will be ours as long as we cultivate that generosity of nature which always prompts to extend the strong arm of help to those in trouble, which eternally beckons to the unfortunate to come and take shelter under it, and ever urges on to the performance of deeds of kindness which are hardly known except to the recipients of them.

Gentlemen, I must bring my remarks to a close.

In this rambling and rather disconnected address, where I am shocked to find the personal pronoun occurring far too frequently, I have discussed questions about which I cannot expect you to take so deep an interest as I do; although I do not doubt your readiness to sympathise with the difficulties which beset a large body of your brethren in the public service.

For my own part, I cannot regard the decadence of the old service to which I once belonged, and to which I owe so much of the success and happiness of life, without some sad reflections. Looking down the long vista of forty years, I recall the memory of old friends, good comrades, skilful surgeons, most of whom have passed away to the great majority. But it is some satisfaction, amidst the threatening wreck, to remember such names as Mouat, Home, Sylvester, Jee, Manly, Reynolds, and others, who have won the Victoria Cross, not by any acts of daring extraneous to their professional calling, but in the holy cause of saving human life—to remember that many were faithful even unto death. How, for example, at that disastrous day at Majuba Hill, a young medical officer, Dr. Landon, was mortally wounded, and laid down to die; and as he lay there, a wounded soldier was brought in suffering great agony. At this supreme moment, this young officer, sore-smitten as he was, ordered the bystanders to prop him up against a rock, and, taking out his hypodermic case, injected with trembling hand some morphia into the arm of his fellow-sufferer. The soldier, eased of his pain, fell into a quiet slumber. The surgeon, shot through the spine, paralysed and dying, slept the sleep that knows no waking. "Inasmuch as ye did it to one of the least of these My brethren, ye did it unto Me."

What the future of the Medical Department of the Army may be I cannot conjecture. But, looking at its past history—at the work it has done in the wars which have taken place since the Crimean campaign, in the Indian Mutiny, in China, Abyssinia, Africa, the Cape, and Egypt—I do not know how men could have done their duty better.

Sir William MacCormac, than whom there can be no better judge of the duties of army-surgeons, writes thus: "In my opinion, the medical officers in the recent Nile campaign displayed the most self-denying devotion to the sick and wounded. There was an exceedingly small mortality. The death rate was only 1·32 per cent. among the sick; among the wounded the mortality was only 3·02 per cent. And the surgical arrangements for the care of the wounded were so successfully carried out, that not a case of infected wounded disease occurred in the hospitals. I know this to be unprecedented in military surgery. At Sedan, under comparatively favourable conditions, so many of the subjects of operation and other cases died of pyæmia, that I felt completely disheartened." So writes one who has had a large experience of war.

But if the history of the past is forgotten, if the bitter lessons taught in the Crimea and Scutari are disregarded, if a mistaken economy, or any other cause, so starves the medical service that highly educated men will not care to adopt it as a career in life, if efficiency and readiness for the exigencies of sudden war are sacrificed for the sake of saving a few thousands, which the nation would never grudge—then will the same disasters recur, and the British soldier will lose the efficient help of that hitherto well-ordered medical organisation which has been ready to share his dangers and to stand by him in the hour of his need.

*Patent certantibus campi. Manat undique cruor. Salus una restat moribundis; ecce chirurgus!*

